# Standard Bread

**Published:** 1923-04

**Authors:** 


### Standard Bread.

SIR,—What has become of Standard Bread ? Before the war, and during the early part of it, this most excellent bread was very largely eaten, and its popularity seemed to be assured. As the war went on, however, it was replaced by a *super* (or was it an *infra* ?) bread which contained a much larger proportion of what are humorously called " milling offals." Most people did not like it, and it did not suit everybody ; I did like it.

When the war was over I expected Standard Bread to return ; but no single loaf of it has ever come my way, and people now try to make me believe that it was merely imposed upon the country by a newspaper " stunt." That is nonsense. The truth I believe to be partly that the bakers can make larger profits by producing semi-baked bread, of a ghastly plaster-of-Paris whiteness, and that great numbers of consumers are so ignorant that they do not know good bread when they see it. I am sure that every dietitian will agree with me that bread out of which all the nutritive properties have been milled is an exceedingly poor food. It is especially undesirable for children, since I take it to be destitute of the constituents which the growing human organism requires. At the present time English white bread is utterly lacking both in flavour and nutriment. Most of our brown bread, on the other hand, is excellent—because, of course, it retains the essential qualities of bread.

